# Dynapenia Rather Than Sarcopenia Is Associated with Metabolic Syndrome in Patients with Chronic Liver Diseases

**DOI:** 10.3390/diagnostics11071262

**Published:** 2021-07-14

**Authors:** Hiroki Nishikawa, Kazunori Yoh, Hirayuki Enomoto, Shuhei Nishiguchi, Hiroko Iijima

**Affiliations:** 1 Department of Internal Medicine, Division of Gastroenterology and Hepatology, Hyogo College of Medicine, Nishinomiya 663-8501, Japan; mm2wintwin@yahoo.co.jp (K.Y.); enomoto@hyo-med.ac.jp (H.E.); hiroko-i@hyo-med.ac.jp (H.I.); 2 The Second Department of Internal Medicine, Osaka Medical and Pharmaceutical University, Osaka 569-8686, Japan; 3 Kano General Hospital, Osaka 531-0041, Japan; nishiguchi@heartfull.or.jp

**Keywords:** chronic liver disease, metabolic syndrome, sarcopenia, dynapenia

## Abstract

We aimed to examine the association between sarcopenia-related factors and metabolic syndrome (Met-S) in patients with chronic liver diseases (CLDs, n = 582, average age = 59.5 years, 290 males, 168 liver cirrhosis cases). Met-S was determined based on the Japanese criteria. Sarcopenia was determined based on grip strength (GS) and skeletal muscle index (SMI) by bioelectrical impedance analysis. Our cohort was divided into the four groups: (A) sarcopenia (n = 44), (B) dynapenia (n = 45), (C) presarcopenia (n = 112), and (D) the control (n = 381). Impacts of GS and SMI on Met-S were investigated. In males, waist circumference (WC) ≥ 85 cm was observed in 199 patients (68.6%), while in females, WC ≥ 90 cm was observed in 94 patients (32.2%). Met-S was identified in 109 patients (18.7%). The proportion of Met-S in the group A, B, C and D were 18.2%, 48.9%, 8.0%, and 18.4% (A vs. B, *p* = 0.0033; B vs. C, *p* < 0.0001; C vs. D, *p* = 0.0081; A vs. C, *p* = 0.0867; A vs. D, *p* = 1.000, B vs. D, *p* < 0.0001; overall *p* value < 0.0001). Multivariate analysis revealed that age, gender, and group B (dynapenia) were significant factors linked to the presence of Met-S. In conclusion, dynapenia rather than sarcopenia is associated with Met-S in CLD patients.

## 1. Introduction

Sarcopenia is a clinical symptom characterized by progressive and generalized decrease of muscle mass and poor muscle function, leading to frailty, cachexia, severe infection, osteoporosis, and thereby all-cause mortality [1,2,3,4,5,6,7]. Daily life inactivity, bedridden status, malnutrition, hormone disorder, and a chronic inflammatory condition are representative clinical features in sarcopenic patients [8]. Alterations in nutritional metabolism, nutritional requirements, and reduced dietary intakes are often encountered in patients with chronic liver diseases (CLDs), and sarcopenia can be also associated with worse patient QOL, poorer prognosis, and more expensive health care costs in patients with CLDs [4,6,7,9]. The interaction between the liver, muscle, and adipose tissue can play a significant role for the development of non-alcoholic fatty liver disease (NAFLD) [10,11]. Sarcopenic obesity can be a risk factor for the development of NAFLD and be linked to liver fibrosis progression [11].

WHO defines metabolic syndrome (Met-S) as a condition characterized by abdominal obesity, higher fasting blood glucose (FBS) level, hyperlipidemia, and hypertension [12]. Patients with Met-S have increased visceral fat and are more prone to cardiovascular diseases, which can be associated with adverse clinical outcomes [13]. Obesity, Met-S, and diabetes mellitus have been increasing in developed countries and will lead to more hepatocellular carcinoma (HCC) cases [14,15]. Met-S can be reversed by appropriate interventions such as exercise training. In 2005, the Japanese Society of Internal Medicine published new diagnostic criteria for Met-S with visceral obesity as the pivotal pathology. Visceral fat mass is closely related to waist circumference (WC). Adipocyte secretes a variety of hormones, commonly known as adipokines and chemokines, and the latter are the ones recruiting macrophages within the adipose tissue, and responsible for TNFα and IL6 secretion [16,17,18].

Choudhary NS, et al. reported the high prevalence of developing Met-S after living donor liver transplantation [19]. Seror M, et al. reported that sarcopenia could be an adverse predictor in patients with Met-S related HCC [20]. However, data for the relevance between sarcopenia-related factors and Met-S in CLD patients remain limited. In the current study, we aimed to examine whether an independent association exists between sarcopenia-related factors and Met-S in patients with CLDs.

## 2. Patients and Methods

### 2.1. Patients

**Figure 1:**
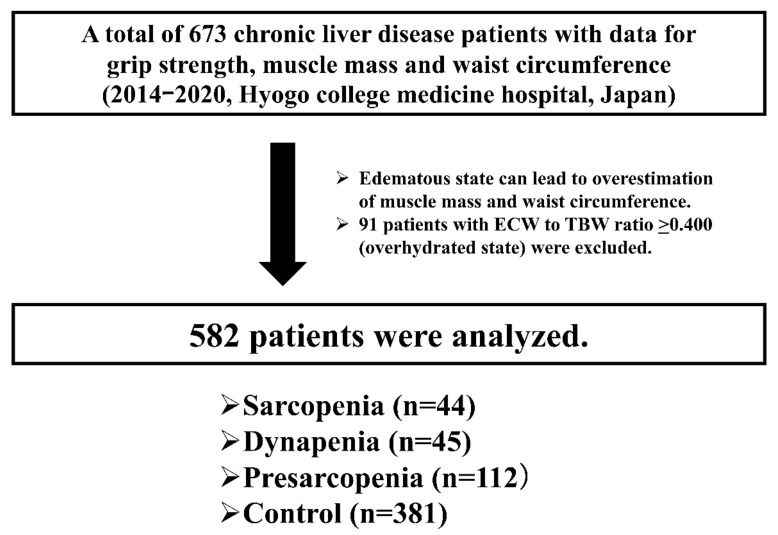
Patient flow chart in this study.

Our hospital involves one of the leading high-volume centers for liver diseases in Japan. Our patient population may not differ significantly from that of major centers in liver disease care in Japan [21,22,23]. In our hospital, anthropometry measurements have been done when patient consent was obtained. The data for each patient have been consecutively recorded in our database. Between January 2014 and October 2020, a total of 682 CLD patients agreed to anthropometry measurements. Of these, 9 cases were excluded because some of the data for grip strength (GS), bioelectrical impedance analysis (BIA), and WC were missing. Thus, there were a total of 673 CLD patients with data for GS, muscle mass using BIA, and WC. All were outpatients with regular visits or inpatients in the Department of Internal Medicine, Division of Gastroenterology and Hepatology, Hyogo College of Medicine Hospital. An expert nutritionist assessed GS, muscle mass using BIA, and WC. BIA can reflect the severity of edematous status (i.e., extracellular water (ECW) to total body water (TBW) ratio). In cases of healthy individuals, ECW to TBW ratio can be maintained at a constant value (around 0.38) [24,25,26]. WC can be overestimated by edematous status. Thus, 91 patients with ECW to TBW ratio ≥ 0.400 (overhydrated state) were excluded from the current analysis, and a total of 582 patients were included into analysis [27] (Figure 1). The patients included in the analysis were not arbitrarily selected. The diagnosis of liver cirrhosis (LC) was based on clinical examination, biochemical, radiographic findings, and/or liver biopsy data.

### 2.2. Definition of Met-S

Met-S was determined based on the current Japanese criteria [28]. WC is a required item for Met-S. Japanese cutoff values of WC are ≥85 cm for males and ≥90 cm for females, and these are defined based on the data from the Japanese Society of Internal Medicine showing that the cutoff values were equivalent to 100 cm^2^ of visceral fat area. In addition to the required item, Met-S is diagnosed if two or more of the following three items are met: hypertriglyceridemia (≥150 mg/dL) and/or low HDL cholesterol (<40 mg/dL), systolic blood pressure ≥130 mmHg, and/or diastolic blood pressure ≥85 mmHg, and FBS ≥110 mg/dL [28]. Patients already receiving pharmacological therapies for hypertriglyceridemia, low HDL cholesterol, hypertension, and diabetes mellitus were included in the relevant category.

### 2.3. Measurement of GS and SMI and Our Study

Muscle strength was assessed by GS according to the current guidelines [3]. Skeletal muscle index (SMI) was defined as “appendicular muscle mass divided by height squared (m^2^)” by BIA. Patients with lower GS were defined as those with GS < 26 kg for males and <18 kg for females [3]. Patients with lower muscle mass were defined as those with SMI < 7.0 kg/m^2^ for males and <5.7 kg/m^2^ for females. Sarcopenia was defined as patients with decreased GS and decreased SMI. Dynapenia was defined as patients with decreased GS and normal SMI [29]. Presarcopenia was defined as patients with normal GS and decreased SMI. Control was defined as patients with normal GS and normal SMI. Thus, our analyzed study subjects were classified into the four categories (Figure 1).

Impacts of sarcopenia-related factors (i.e., GS and SMI) on Met-S were investigated for all cases and several subgroups according to the LC status, age, gender, and body mass index (BMI). Factors associated with the presence of Met-S were also examined using univariate and multivariate analyses. We received the ethical approval from ethics committee of Hyogo college of medicine hospital. The protocol in this study strictly followed all regulations of the 1975 Declaration of Helsinki.

### 2.4. Statistical Considerations

In terms of continuous parameters, Student’s *t* test or Mann-Whitney *U* test was applied to estimate between-group difference, as appropriate. Data for continuous parameters were presented as average value ± standard deviation (SD). In terms of categorical parameters, Fisher’s exact tests or Pearson χ^2^ test was applied to estimate between-group difference, as appropriate. Factors with *p* < 0.05 linked to the presence of Met-S in the univariate analysis were entered into the multivariate logistic regression analysis to identify independent factors. The cutoff point for statistical significance was set at *p* = 0.05 by using the JMP 14.1 (SAS Institute Inc., Cary, NC, USA).

## 3. Results

### 3.1. Patient Baseline Characteristics

Baseline characteristics for all cases (n = 582, 290 males and 292 females, average age = 59.5 years) are presented in Table 1. LC was found in 168 patients (28.9%). The average (±SD) ECW to TBW ratio was 0.386 ± 0.007. The average (±SD) WC for males and females were 90.1 ± 9.8 cm and 86.5 ± 11.5 cm, respectively. In males, WC ≥ 85 cm was observed in 199 patients (68.6%), while in females, WC ≥ 90 cm was observed in 94 patients (32.2%). BMI ≥ 25 kg/m^2^ (overweight or obese as defined by WHO criteria) was found in 105 patients (36.2%) for males and 83 patients (28.4%) for females. GS decline was found in 29 patients (10.0%) for males and 60 patients (20.5%) for females. SMI decline was found in 68 patients (23.4%) for males and 88 patients (30.1%) for females. Hypertriglyceridemia (≥150 mg/dL) and/or low HDL cholesterol (<40 mg/dL) was identified in 198 patients (34.0%). Systolic blood pressure ≥ 130 mmHg and/or diastolic blood pressure ≥85 mmHg was identified in 183 patients (31.4%). Hyperglycemia (FBS ≥ 110 mg/dL) was identified in 203 patients (34.9%). Met-S was identified in 109 patients (18.7%: 68 patients (23.5%) for males and 41 patients (14.0%) for females). Overall, there were 44 patients in the sarcopenia group, 45 in the dynapenia group, 112 in the presarcopenia group, and 381 in the control group. Baseline characteristics in the 4 groups are presented in Table 1.

### 3.2. Proportion of Met-S for All Cases Stratified by GS and SMI

**Figure 2:**
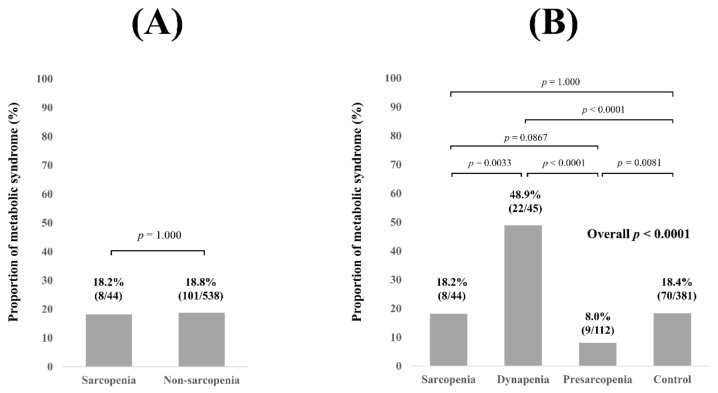
(**A**) Proportion of Met-S in patients with sarcopenia and non-sarcopenia for all cases (n = 582). (**B**) Proportion of Met-S in patients with sarcopenia, dynapenia, presarcopenia, and control for all cases.

No significant difference of the proportion of Met-S was observed in comparison of sarcopenia and non-sarcopenia (i.e., the dynapenia group, the presarcopenia group, and the control group) (*p* = 1.000, Figure 2A). Overall significance for the proportion of Met-S was found among the 4 groups (overall *p* value < 0.0001). *P* values between any two groups are demonstrated in Figure 2B.

### 3.3. Subgroup Analysis 1: Proportion of MetS Stratified by GS and SMI According to the LC Status

**Figure 3:**
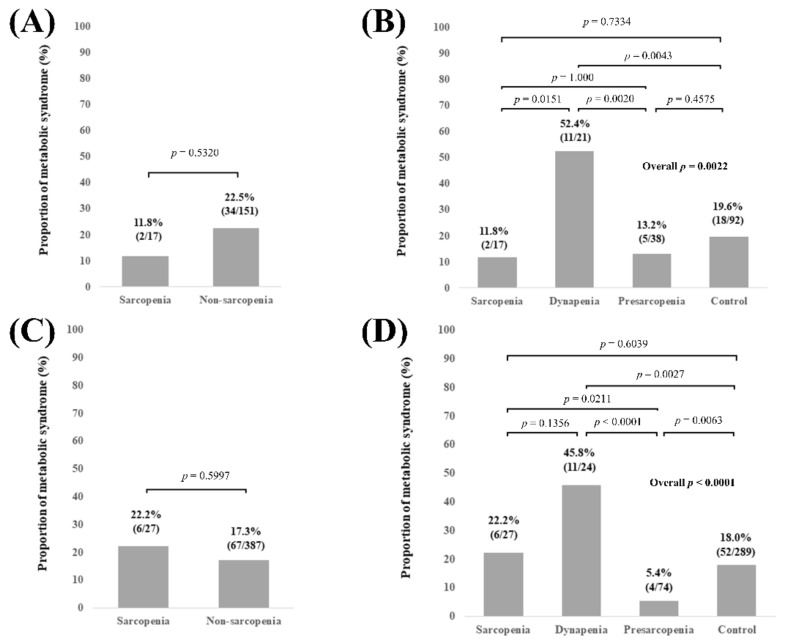
(**A**) Proportion of Met-S in patients with sarcopenia and non-sarcopenia in LC patients (n = 168). (**B**) Proportion of Met-S in patients with sarcopenia, dynapenia, presarcopenia, and control in LC patients. (**C**) Proportion of Met-S in patients with sarcopenia and non-sarcopenia in non-LC patients (n = 414). (**D**) Proportion of Met-S in patients with sarcopenia, dynapenia, presarcopenia, and control in non-LC patients.

In LC patients (n = 168), no significant difference of the proportion of MetS was observed in comparison of sarcopenia and non-sarcopenia (*p* = 0.5320, Figure 3A). Overall significance for the proportion of MetS was found among the 4 groups (overall *p* value = 0.0022). *p* values between any two groups are demonstrated in Figure 3B. In non-LC patients (n = 414), no significant difference of the proportion of MetS was observed in comparison of sarcopenia and non-sarcopenia (*p* = 0.5997, Figure 3C). Overall significance for the proportion of MetS was found among the 4 groups (overall *p* value < 0.0001). *p* values between any two groups are demonstrated in Figure 3D.

### 3.4. Subgroup Analysis 2: Proportion of Met-S Stratified by GS and SMI According to Age

**Figure 4:**
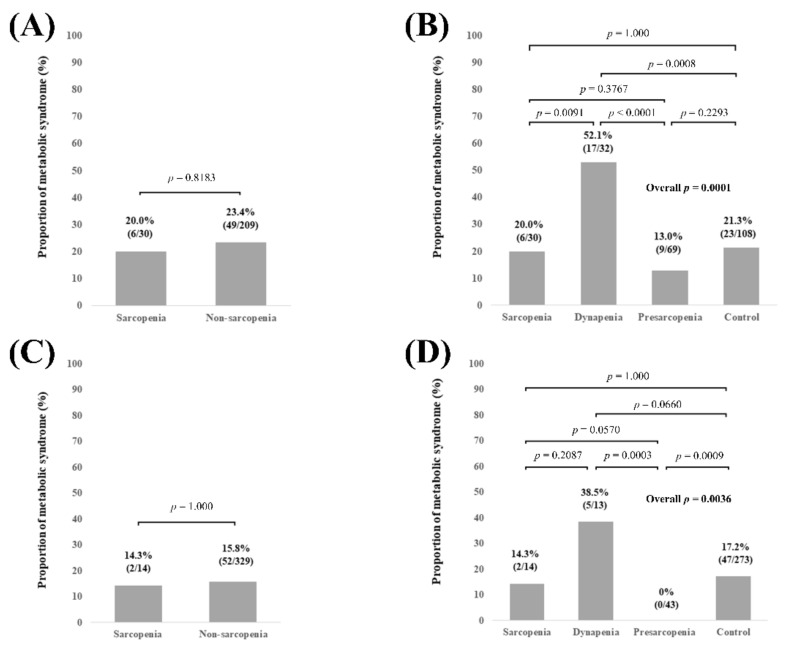
(**A**) Proportion of Met-S in patients with sarcopenia and non-sarcopenia in patients aged 65 years or more (n = 239). (**B**) Proportion of Met-S in patients with sarcopenia, dynapenia, presarcopenia, and control in patients aged 65 years or more. (**C**) Proportion of Met-S in patients with sarcopenia and non-sarcopenia in patients less than 65 years (n = 343). (**D**) Proportion of Met-S in patients with sarcopenia, dynapenia, presarcopenia, and control in patients less than 65 years.

In patients aged 65 years or more (n = 239), no significant difference of the proportion of Met-S was observed in comparison of sarcopenia and non-sarcopenia (*p* = 0.8183, Figure 4A). Overall significance for the proportion of Met-S was found among the 4 groups (overall *p* value = 0.0001). *p* values between any two groups are demonstrated in Figure 4B. In patients aged less than 65 years (n = 343), no significant difference of the proportion of Met-S was observed in comparison of sarcopenia and non-sarcopenia (*p* = 1.000, Figure 4C). Overall significance for the proportion of Met-S was found among the 4 groups (overall *p* value = 0.0036). *p* values between any two groups are demonstrated in Figure 4D.

### 3.5. Subgroup Analysis 3: Proportion of Met-S Stratified by GS and SMI According to Gender

**Figure 5:**
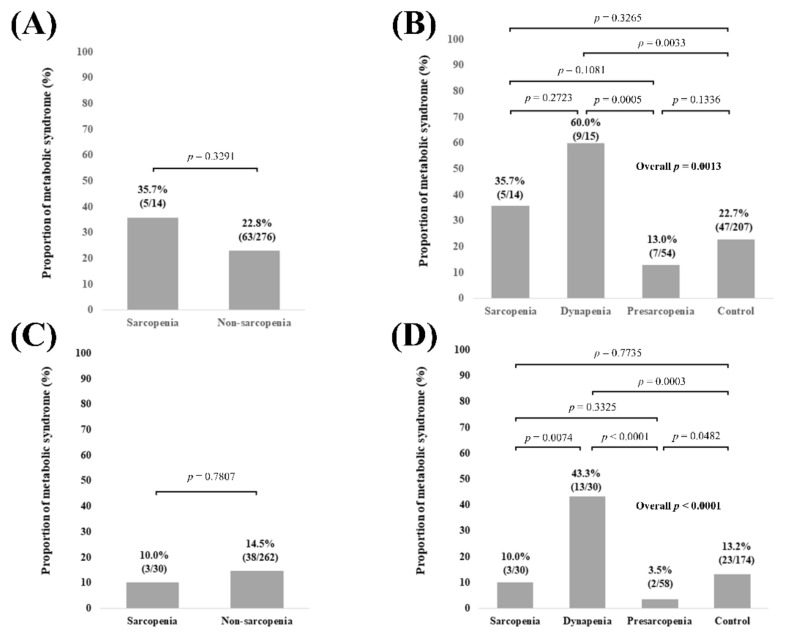
(**A**) Proportion of Met-S in patients with sarcopenia and non-sarcopenia in male patients (n = 290). (**B**) Proportion of Met-S in patients with sarcopenia, dynapenia, presarcopenia, and control in male patients. (**C**) Proportion of Met-S in patients with sarcopenia and non-sarcopenia in female patients (n = 292). (**D**) Proportion of Met-S in patients with sarcopenia, dynapenia, presarcopenia, and control in female patients.

In male patients (n = 290), no significant difference of the proportion of Met-S was observed in comparison of sarcopenia and non-sarcopenia (*p* = 0.3291, Figure 5A). Overall significance for the proportion of Met-S was found among the 4 groups (overall *p* value = 0.0013). *p* values between any two groups are demonstrated in Figure 5B. In female patients (n = 292), no significant difference of the proportion of Met-S was observed in comparison of sarcopenia and non-sarcopenia (*p* = 0.7807, Figure 5C). Overall significance for the proportion of Met-S was found among the 4 groups (overall *p* value < 0.0001). *p* values between any two groups are demonstrated in Figure 5D.

### 3.6. Subgroup Analysis 4: Proportion of Met-S Stratified by GS and SMI According to BMI

**Figure 6:**
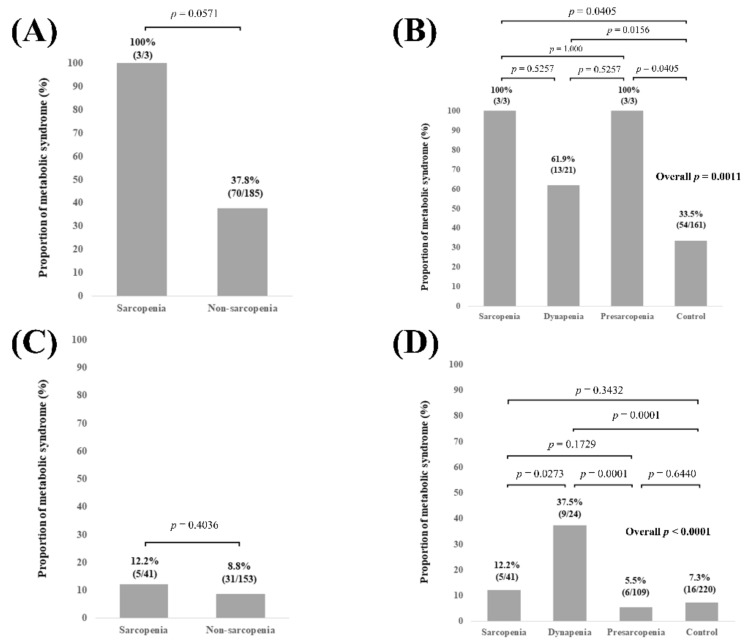
(**A**) Proportion of Met-S in patients with sarcopenia and non-sarcopenia in patients with BMI ≥ 25 kg/m^2^ (n = 188). (**B**) Proportion of Met-S in patients with sarcopenia, dynapenia, presarcopenia, and control in patients with BMI ≥ 25 kg/m^2^. (**C**) Proportion of Met-S in patients with sarcopenia and non-sarcopenia in patients with BMI < 25 kg/m^2^ (n = 394). (**D**) Proportion of Met-S in patients with sarcopenia, dynapenia, presarcopenia, and control in patients with BMI < 25 kg/m^2^.

In patients with BMI ≥ 25 kg/m^2^ (n = 188), no significant difference of the proportion of Met-S was observed in comparison of sarcopenia and non-sarcopenia (*p* = 0.0571, Figure 6A). Overall significance for the proportion of Met-S was found among the 4 groups (overall *p* value = 0.0011). *p* values between any two groups are demonstrated in Figure 6B. In patients with BMI < 25 kg/m^2^ (n = 394), no significant difference of the proportion of Met-S was observed in comparison of sarcopenia and non-sarcopenia (*p* = 0.4036, Figure 6C). Overall significance for the proportion of Met-S was found among the 4 groups (overall *p* value < 0.0001). *p* values between any two groups are demonstrated in Figure 6D.

### 3.7. Univariate and Multivariate Analyses of Factors Associated with the Presence of Mets

According to the criteria of WHO for Mets, BMI is included in the definition of Mets, and BMI was not entered into the analysis. Age (*p* = 0.0040), gender (*p* = 0.0041), our classification stratified by GS and SMI (*p* < 0.0001), and estimated glomerular filtration rate (*p* = 0.0026) were significant factors linked to the presence of Met-S in the univariate analysis. (Table 2) Age (*p* = 0.0438), gender (*p* = 0.0003), and dynapenia (*p* = 0.0001, control as a reference) were significant factors linked to the presence of Met-S in the multivariate analysis. (Table 3) Hazard ratios (HR) and 95% confidence intervals (CI) in each factor are shown in Table 3.

## 4. Discussion

In the field of public health, Met-S has been attracting much caution these days due to its prognostic significance, and the same is true for sarcopenia. Met-S is a constellation of risk factors that will often result in increased severity of metabolic defects if left untreated [30]. A recent meta-analysis demonstrated that Met-S was associated with a moderately increased risk of liver-related adverse events, and patients with Met-S together with hepatitis B viral infection are more likely to develop liver-related adverse events [31]. Dynapenic and abdominal obese elderly persons may be at greater risk of metabolic alterations than those with dynapenia alone or those with neither dynapenia nor abdominal obesity [32]. Sarcopenic status with obesity (i.e., sarcopenic obesity) is also a well-established disease entity [33]. However, association between sarcopenia-related factors and Met-S in CLD patients remains to be unsolved. We thus performed the current analysis.

In our results, the frequency of Met-S in dynapenia was the highest for all cases and all subgroups except for cases with BMI ≥ 25 kg/m^2^ among the four groups, and dynapenia was an independent factor associated with the presence of Met-S in the multivariate analysis (HR = 4.020, 95% CI = 1.989–8.125, *p* = 0.0001, the control group as a reference). These results denoted that dynapenia rather than sarcopenia is associated with Met-S in patients with CLDs. In other words, muscle function rather than muscle mass can be a key factor for Met-S. The facts that sarcopenic patients tended to have a lower WC and dynapenic patients tended to have a higher WC are closely related to the present results. Even in CLD patients with preserved muscle mass without any loss of body weight, muscle weakness should be noted. There were only 3 sarcopenic obesity (BMI ≥ 25 kg/m^2^) patients (0.5%), whereas there were 21 dynapenic obesity patients (3.6%) in our cohort, which may also be linked to the current results. On the other hand, the proportion of patients with baseline fasting blood glucose ≥110 mg/dL was the highest in the dynapenia group among the four groups, followed by the sarcopenia group (40.9% (18/44) in the sarcopenia group, 51.1% (23/45) in the dynapenia group, 33.0% (37/112) in the presarcopenia group, and 32.8% (125/381) in the control group). Thus, insulin resistance may be associated with muscle strength rather than muscle mass [34].

GS measurement was reported to be a simple, inexpensive risk stratification method for all-cause mortality, cardiovascular-related death, and cardiovascular diseases in a large observational study [35]. An improvement of GS significantly reduced the risk of cancer-related mortality [36]. A GS decrease was reported to be closely associated with liver-related adverse events in CLD patients [37]. A decline in muscle strength before the decline of muscle mass is proposed to be “dynapenia” [29,38]. Indeed, dynapenia is a very important clinical entity, as well as sarcopenia. Our current results may not be surprising in a sense, given that patients with dynapenia have a relatively higher BMI and WC because their muscle mass is preserved. Yang et al. presented that dynapenic obesity was associated with an elevated risk of disability compared with dynapenia alone or obesity alone in 616 community-dwelling elder people [39]. A previous Italian observational study reported that dynapenic and abdominal obese patients were at elevated risk of worsening disability and mortality than patients with dynapenia alone or abdominal obesity alone [40]. Thus, in CLD patients with both dynapenia and Met-S, we should pay special attention for developing disability. On the other hand, it should be noted that the prevalence of Met-S in patients with presarcopenia tended to be lower than other 3 groups. Decrease of muscle mass itself may not influence on metabolic status in CLD patients.

Under normal condition, skeletal muscle is responsible for the majority of insulin-stimulated systemic glucose processing, and thus metabolic abnormalities in skeletal muscle can strongly influence glucose homeostasis and insulin sensitivity [41]. Skeletal muscle disorders in type 2 diabetic patients are predominantly dynapenia rather than sarcopenia; sarcopenia occurs more frequently in patients with a low BMI, whereas dynapenia occurs more frequently in patients with a high BMI, which may have contributed to the present results [42]. Mori et al., reported that in 166 type 2 diabetic patients, sarcopenia and dynapenia were observed in 7.2% and 13.9%, while in our 204 patients with FBS ≥ 110 mg/dL, 18 (8.8%) had sarcopenia and 23 (11.3%) had dynapenia, which is in agreement with the data by Mori, et al. [42]. On the other hand, according to statistics in recent years from the Japanese Ministry of Health, Labor, and Welfare, the frequency of Met-S in Japanese adults has been reported to be around 25–30% for males and around 10% for females. While in our data, 68 patients (23.5%) for males and 41 patients (14.0%) for females had Met-S, and male was an independent factor linked to the presence of Met-S in our multivariate analysis, which is not largely different from the data in Japanese adults. CLDs themselves may not affect the frequency of Met-S.

Several limitations must be mentioned in the present study. First, our study was a single-center cross-sectional study with a retrospective nature. Thus, the researchers’ subjectivity in patient selection cannot be excluded. Second, GS can vary according to patient daily life activities. Third, WC can be overestimated by the edematous status, and thus patients with ECW to TBW ratio ≥ 0.4 were excluded from this analysis. We believe that this exclusion was adequate, but patients with ECW to TBW ratio ≥ 0.4 are more likely to involve advanced LC status with the high possibility of having sarcopenia. Our current data may not be applied in patients with advanced LC status. Finally, the causal relationship between Met-S and sarcopenia-related factors was unclear due to the cross-sectional nature of our study. Caution should be therefore paid for the interpretation of our data. Despite the limitations, our study results demonstrated that not muscle mass, but muscle function was closely associated with Met-S in CLD patients. In conclusion, dynapenia rather than sarcopenia is associated with Met-S in patients with CLDs.

## Figures and Tables

**Table 1:** Baseline characteristics (n = 582).

Variables	All Cases (n = 582)	Sarcopenia (n = 44)	Dynapenia (n = 45)	Presarcopenia (n = 112)	Control (n = 381)
Age (years)	59.5 ± 12.6	66.4 ± 11.9	67.6 ± 9.1	64.8 ± 11.4	56.2 ± 12.1
Gender, male/female	290/292	14/30	15/30	54/58	207/174
Liver disease etiologyHBV/HCV/others	89/255/238	5/12/17	4/23/18	19/56/37	61/154/166
Presence of LC, yes/no	168/414	17/27	21/24	38/74	92/289
Body mass index (kg/m^2^)	23.7 ± 4.1	20.8 ± 1.8	25.0 ± 2.6	20.1 ± 2.1	24.9 ± 4.1
Grip strength (kg), male	35.8 ± 7.6	24.0 ± 1.7	21.9 ± 4.9	31.8 ± 4.5	38.6 ± 6.1
Grip strength (kg), female	22.1 ± 5.1	15.2 ± 2.9	15.7 ± 2.9	21.8 ± 2.4	24.4 ± 4.3
Skeletal muscle index (kg/m^2^), male	7.6 ± 0.8	6.4 ± 0.4	7.7 ± 0.5	6.5 ± 0.3	8.0 ± 0.7
Skeletal muscle index (kg/m^2^), female	6.1 ± 0.8	5.2 ± 0.4	6.3 ± 0.6	5.3 ± 0.3	6.5 ± 0.6
Waist circumference (cm), male	90.1 ± 9.8	84.5 ± 5.4	93.6 ± 5.4	82.7 ± 7.0	92.2 ± 9.8
Waist circumference (cm), female	86.5 ± 11.5	81.0 ± 7.2	92.4 ± 9.3	77.9 ± 7.4	89.3 ± 11.7
ECW to TBW ratio	0.386 ± 0.007	0.392 ± 0.005	0.390 ± 0.006	0.389 ± 0.005	0.385 ± 0.007
Total bilirubin (mg/dL)	0.9 ± 0.6	0.8 ± 0.4	1.0 ± 0.6	0.9 ± 0.4	0.9 ± 0.6
Serum albumin (g/dL)	4.2 ± 0.4	4.0 ± 0.6	4.0 ± 0.4	4.2 ± 0.4	4.2 ± 0.4
Prothrombin time (INR)	1.06 ± 0.13	1.08 ± 0.12	1.09 ± 0.10	1.07 ± 0.18	1.05 ± 0.12
Platelet count (×10^4^/mm^3^)	18.7 ± 7.6	16.5 ± 7.0	15.1 ± 6.5	17.8 ± 7.4	19.6 ± 7.7
Hypertriglyceridemia (≥150 mg/dL) and/or low HDL (<40 mg/dL), yes/no	198/384	23/21	24/21	32/78	119/262
Fasting blood glucose ≥ 110 mg/dL, yes/no	203/379	18/26	23/22	37/75	125/256
Systolic blood pressure ≥ 130 mmHg and/or diastolic blood pressure ≥85 mmHg, yes/no	183/399	19/25	25/20	35/77	104/277
Metabolic syndrome, yes/no	109/473	8/36	22/23	9/103	70/311
AST (IU/L)	35.9 ± 42.2	35.1 ± 21.2	40.3 ± 26.6	31.8 ± 20.3	36.6 ± 49.6
ALT (IU/L)	37.4 ± 54.7	33.6 ± 40.7	40.4 ± 40.2	27.9 ± 24.1	40.2 ± 63.2
eGFR (ml/min/1.73 m^2^)	83.3 ± 21.4	79.6 ± 26.1	77.8 ± 22.2	80.8 ± 22.9	85.1 ± 20.0

Data are expressed as number or mean value (± standard deviation). HBV: hepatitis B virus, HCV: hepatitis C virus, LC: liver cirrhosis, ECW: extracellular water, TBW: total body water, AST: aspartate aminotransferase, ALT: alanine aminotransferase, eGFR: estimated glomerular filtration rate.

**Table 2:** Univariate analyses of factors linked to the presence of metabolic syndrome.

Variables	Metabolic Syndrome, Yes (n = 109)	Metabolic Syndrome, No (n = 473)	*p* Value
Age (years)	65 (55, 71)	61 (49.5, 68)	0.0040
Gender, male/female	68/41	222/251	0.0041
HBV/HCV/others	19/37/53	70/218/185	0.0692
Presence of LC, yes/no	36/73	132/341	0.2931
Our classification by GS and SMI	8/22/9/70	36/23/103/311	<0.0001
Total bilirubin (mg/dL)	0.8 (0.6, 1.0)	0.8 (0.6, 1.0)	0.9473
Serum albumin (g/dL)	4.2 (3.95, 4.5)	4.2 (4.0, 4.5)	0.9510
Prothrombin time (INR)	1.03 (0.97, 1.095)	1.03 (0.98, 1.09)	0.7913
Platelet count (×10^4^/mm^3^)	17.8 (12.55, 23.1)	18.8 (13.1, 23.35)	0.2648
AST (IU/l)	30 (21.5, 47)	25 (20, 37)	0.5935
ALT (IU/l)	30 (18, 56)	22 (15, 40)	0.1778
ECW to TBW ratio	0.388 (0.383, 0.393)	0.387 (0.381, 0.392)	0.1475
eGFR (ml/min/1.73 m^2^)	78 (66, 89.5)	82 (71, 97)	0.0026

Data are expressed as number or median (interquartile range). HBV: hepatitis B virus, HCV: hepatitis C virus, LC: liver cirrhosis, GS: grip strength, SMI: skeletal muscle index, AST: aspartate aminotransferase, ALT: alanine aminotransferase, ECW: extracellular water, TBW: total body water, eGFR: estimated glomerular filtration rate.

**Table 3:** Multivariate analyses of factors linked to the presence of metabolic syndrome.

	Multivariate Analysis		
	Hazard Ratio	95% CI	*p* Value
Age (per one year)	1.023	1.001–1.046	0.0438
Gender (male)	2.361	1.482–3.760	0.0003
eGFR (per one ml/min/1.73 m^2^)	0.989	0.977–1.001	0.0623
Our classification by GS and SMI			
Sarcopenia	0.879	0.371–2.084	0.7703
Dynapenia	4.020	1.989–8.125	0.0001
Presarcopenia	0.350	0.110–1.001	0.0521
Control	1.000	Reference	

BMI: body mass index, eGFR: estimated glomerular filtration rate, GS: grip strength, SMI: skeletal muscle index, CI: confidence interval.

## Data Availability

The data are not publicly available for the viewpoint of protecting personal information.

## Abbreviations

CLD: chronic liver disease, NAFLD: non-alcoholic fatty liver disease, Met-S: metabolic syndrome, FBS: fasting blood glucose, HCC: hepatocellular carcinoma, WC: waist circumference, GS: grip strength, BIA: bioelectrical impedance analysis, ECW: extracellular water, TBW: total body water, LC: liver cirrhosis, SMI: skeletal muscle index, BMI: body mass index, SD: standard deviation, HR: hazard ratio, CI: confidence interval.
